# Accumulation of Ag(I) by *Saccharomyces cerevisiae* Cells Expressing Plant Metallothioneins

**DOI:** 10.3390/cells7120266

**Published:** 2018-12-11

**Authors:** Lavinia L. Ruta, Melania A. Banu, Aurora D. Neagoe, Ralph Kissen, Atle M. Bones, Ileana C. Farcasanu

**Affiliations:** 1 Faculty of Chemistry, University of Bucharest, Sos. Panduri 90-92, 050663 Bucharest, Romania; lavinia.ruta@chimie.unibuc.ro; 2 Faculty of Biology, University of Bucharest, Splaiul Independentei 91-95, 050095 Bucharest, Romania; melania.banu@imt.ro (M.A.B.); auroradaniela.neagoe@g.unibuc.ro (A.D.N.); 3 Cell, Molecular Biology and Genomics Group, Department of Biology, Norwegian University of Science and Technology, NO-7491 Trondheim, Norway; ralph.kissen@ntnu.no (R.K.); atle.bones@bio.ntnu.no (A.M.B.)

**Keywords:** silver, metallothionein, *Arabidopsis thaliana*, *Noccaea caerulescens*, *Saccharomyces cerevisiae*, accumulation

## Abstract

The various applications of Ag(I) generated the necessity to obtain Ag(I)-accumulating organisms for the removal of surplus Ag(I) from contaminated sites or for the concentration of Ag(I) from Ag(I)-poor environments. In this study we obtained Ag(I)-accumulating cells by expressing plant metallothioneins (MTs) in the model *Saccharomyces cerevisiae*. The cDNAs of seven *Arabidopsis thaliana* MTs (AtMT1a, AtMT1c, AtMT2a, AtMT2b, AtMT3, AtMT4a and AtMT4b) and four *Noccaea caerulescens* MTs (NcMT1, NcMT2a, NcMT2b and NcMT3) fused to myrGFP displaying an *N*-terminal myristoylation sequence for plasma membrane targeting were expressed in *S. cerevisiae* and checked for Ag(I)-related phenotype. The transgenic yeast cells were grown in copper-deficient media to ensure the expression of the plasma membrane high-affinity Cu(I) transporter Ctr1, and also to elude the copper-related inhibition of Ag(I) transport into the cell. All plant MTs expressed in *S. cerevisiae* conferred Ag(I) tolerance to the yeast cells. Among them, myrGFP-NcMT3 afforded Ag(I) accumulation under high concentration (10–50 μM), while myrGFP-AtMT1a conferred increased accumulation capacity under low (1 μM) or even trace Ag(I) (0.02–0.05 μM). The ability to tolerate high concentrations of Ag(I) coupled with accumulative characteristics and robust growth showed by some of the transgenic yeasts highlighted the potential of these strains for biotechnology applications.

## 1. Introduction

Contamination with heavy metals represents a world-wide concern constantly prompting the necessity to develop cost-effective eco-friendly technologies for the remediation of polluted sites [1]. Among the metal contaminants, silver is highly toxic especially in aquatic environments [2], where silver in all forms—free cationic Ag(I), colloidal or nanoparticles (AgNP)—exerts deleterious effects towards living organisms [2,3]. The sources of silver in the environment are diverse, as silver is widely used in modern industry, medicine, aerospace, catalysis, photonics, optoelectronics, etc. [4]. Numerous studies emphasized the need to develop biotechnologies for the remediation of environmental matrices contaminated by heavy metals. In this context, engineered yeasts have biotechnological potential for metal removal and/or recovery [5,6,7,8,9].

The prerequisite for efficient bioremediation techniques is the development of organisms modified to accumulate the contaminant without developing the inherent toxicity symptoms. Recently, we found that *Saccharomyces cerevisiae* cells expressing plant metallothioneins (MTs) targeted to the cytosolic face of the plasma membrane accumulate divalent metal cations such as Cd(II), Co(II), Cu(II), Mn(II), or Ni(II) [10]. MTs are metal-binding proteins found across most taxonomic groups involved in the heavy metal tolerance of many eukaryotes, including yeasts, mammals, and plants [11]. Being cysteine-rich proteins (close to 30% of their amino acid content), they tend to form metal-thiolate complexes based on metal ion coordination, and while some roles still remain obscure, it is widely accepted that all MTs have an undisputed capacity to buffer intracellular metal ions, especially Zn(II) and Cu(I) [12]. Based on their innate metal-binding abilities, MTs are classified into Cu(I)- and Zn(II)-thioneins, with the representative non-essential counterparts Ag(I) and Cd(II), respectively [12,13]. Considering the affinity of heavy metal cations for thiolate ligands, it was shown that Ag(I) follows Cu(I) in this affinity series, with Ag(I) exhibiting lower affinity [14]; this is why it is expected that Cu(I) would be preferably bound by metallothioneins when both cations are present. The use of heterologous expression of metallothioneins to obtain heavy metal accumulating organisms is widely encountered and *S. cerevisiae* cells are often used as eukaryotic microorganism hosts [15]. While *S. cerevisiae*-expressing plant metallothioneins were shown to be tolerant to and to accumulate a variety of heavy metals [15], studies on Ag(I) accumulation are scarce [16]. In this study, we investigated the possibility to obtain silver-accumulating cells by heterologous expression of plant MTs targeted to the inner face of the yeast plasma membrane. Differently from our previous study, in which recombinant plant MTs overlapped the innate MT (ScMT or Cup1) [10], in the present study the plant MTs were expressed in yeast cells not expressing their innate MT, Cup1.

## 2. Materials and Methods

### 2.1. Yeast Strains and Growth Media

The *S. cerevisiae* strains used in this study were isogenic with the “wild-type” (WT) parental strain BY4741 (*MAT***a**; *his3Δ1*; *leu2Δ0*; *met15Δ0*; *ura3Δ0*) [17]. The knockout mutant strains used were Y05539 (BY4741, *ctr1::kanMX4*), Y04533 (BY4741, *cup2::kanMX4*), Y06461 (BY4741, *fet4::kanMX4*), Y06272 (BY4741, *smf1::kanMX4*), and Y06524 (BY4741, *pho84::kanMX4*), denoted *ctr1Δ, cup2Δ*, *fet4Δ*, *smf1Δ* and *pho84Δ*, respectively. The strains were obtained from EUROSCARF (European *S. cerevisiae* Archive for Functional Analysis, www.euroscarf.de) and were propagated, grown, and maintained in YPD medium (1% yeast extract, 2% polypeptone, 2% glucose) or SD (0.17% yeast nitrogen base without amino acids, 0.5% (NH_4_)_2_SO_4_, 2% glucose, supplemented with the necessary amino acids) [18]. The strains transformed with the plasmids harboring MT cDNA-s [10] were selected and maintained on SD lacking uracil (SD-Ura). For induction of MT cDNA expression, cells were pre-grown in synthetic medium containing 2% raffinose (SRaf-Ura) before being shifted to galactose-containing media [19]. Minimal defined media (MM) [18] were prepared adding individual components as described [18] using ultrapure reagents (Merck, Darmstadt, Germany). MM were prepared in acid-washed glasswear to ensure controlled metal concentrations. As carbon source, MM could contain 2% glucose (MM/Glc), 2% galactose (MM/Gal) or 2% Raf (MM/Raf), as necessary. MM media thus prepared were virtually Ag(I)-free and contained 0.25 µM Cu(II). To obtain copper-free MM, or copper dropout MM, CuSO_4_ was omitted from the recipe; the absence of copper was confirmed by ICP-MS. Minimal medium with low copper (MMLC) contained 0.1 µM Cu(II). All synthetic media had their pH adjusted to 6. For solid media, 2% agar was used. For growth improvement, all the synthetic media were supplemented with an extra 20 mg/L leucine [20].

### 2.2. Plasmids and Yeast Transformation

For heterologous expression of plant MTs, yeast cells were transformed with *URA3*-based plasmids harboring cDNAs of *Arabidopsis thaliana* (AtMT1a, AtMT1c, AtMT2a, AtMT2b, AtMT3, AtMT4a, and AtMT4b) and *Noccaea caerulescens* (NcMT1, NcMT2a, NcMT2b, and NcMT3) MTs, fused to myrGFP (GFP displaying an *N*-terminal myristoylation sequence) [10]. The resulting fusion proteins are generally referred to as myrGFP-MT in the text. The cDNAs were under the control of *GAL1* promoter, allowing strong induction of cDNA expression when cells are shifted to media containing galactose as sole carbon source [19]. Yeast transformation [21] was performed using S.c. EasyComp™ Transformation Kit (Invitrogen, Catalog number: K505001) following manufacturer’s indications.

### 2.3. Yeast Cell Growth Assay

#### 2.3.1. Growth in Liquid Media

Wild-type BY4741 yeast cells were pre-grown overnight in SRaf then diluted in fresh SRaf medium to density 5 × 10^5^ cells/mL. Cells were grown to 1 × 10^6^ cells/mL then shifted to MM/Gal for proliferation assay under various conditions. The growth conditions presented above were applied to WT for the sake of uniformity, as strains expressing MTs had to be grown in media containing galactose as carbon source, for transgene induction. Yeast expressing recombinant MTs were grown overnight in SRaf-Ura and inoculated in fresh MM/Raf-Ura to 5 × 10^5^ cells/mL. Cells were grown to 1 × 10^6^ cells/mL. At this point (considered time 0), cells were harvested, and shifted to minimal media containing galactose (MM/Gal-Ura) for transgene induction. Cell growth in liquid media was monitored by measuring culture turbidity at 660 nm (OD_660)_ [22], recorded by a plate reader equipped with thermostat and shaker (Varioskan, Thermo Fisher Scientific, Vantaa, Finland). When used, surplus metal ions were added from sterile stocks of ultra-pure 0.1 M AgNO_3_ or 0.1 M CuSO_4_. Unless otherwise stated, all incubation was done with agitation (200 rpm) at 30 °C. Before shifting to galactose-media, the cell viability was checked by staining with methylene blue (Roth, Karsruhe, Germany) and only populations with viability >99% were used further.

#### 2.3.2. Growth on Solid Media

For growth on solid media, the transformed cells pre-grown in SRaf-Ura were shifted to SGal-Ura and grown for 6 h for transgene induction. Cells were 10-fold serially diluted in a 48-well microtiter plate and stamped on SGal-Ura agar plates using a pin replicator (approximately 4 μL/spot). Plates were photographed after incubation at 30 °C for 3 days.

#### 2.3.3. Cell Viability and LC_50_

The cell viability, expressed as percentage of live cells within a whole population, was assessed by staining with methylene blue. Viability was examined for at least 300 cells from one biological replicate. Viable cells were colorless, and dead cells were blue. Original cell suspensions (grown in normal medium) had a viability higher than 99%. Lethal concentration 50 (LC_50_) represents the Ag(I) concentration eliciting a 50% reduction in cell proliferation after 24 h of incubation in the presence of various Ag(I) concentrations and it was determined by probit analysis for each strain.

### 2.4. Silver Accumulation by Growing Cells

“Wild-type” BY4741 yeast cells were pregrown in SRaf medium then shifted to MM/Gal medium and grown to density 5 × 10^6^ cells/mL before Ag(I) was added to the desired concentration. Similarly, cells transformed with recombinant MT cDNAs and the myrGFP control vector were pregrown in SRaf-Ura then shifted to MM/Gal-Ura for transgene induction and grown to density 5 × 10^6^ cells/mL before Ag(I) was added to the desired concentration. Ag(I) was added from a sterile 0.1 M AgNO_3_ stock. To measure the metal accumulated, cells were harvested by centrifugation (1 min, 5000 rpm, 4 °C) and washed three times with ice-cold 10 mM 2-(*N*-morpholino)-ethanesulfonic acid (MES)-Tris buffer containing 10 mM KCl, pH 6.0, before being suspended in deionized water (final density, 10^8^ cells/mL). For metal assay, cells were digested with 65% ultrapure HNO_3_ (Merck, Darmstadt, Germany). Metal analysis was done using an instrument with a single collector, quadrupole inductively coupled plasma with mass spectrometry (ICP–MS, Perkin-Elmer ELAN DRC-e, Concord, ON, Canada) against Multielement ICP Calibration Standard 3, matrix 5% HNO_3_ (Perkin Elmer Pure Plus). The metal cellular content was normalized to total cellular proteins, assayed spectrophotometrically [23]. Values were expressed as the mean ± standard error of the mean (SEM) of triplicate determinations on three independent yeast transformants.

### 2.5. myrGFP-MT Localization

For the detection of myrGFP-MT fluorescence, the transformed cells grown overnight in SRaf-Ura were shifted to MM/Gal-Ura for transgene induction and grown for 4–6 h before being visualized by fluorescence microscopy. Live cells were examined with an Olympus fluorescent microscope system (Olympus BX53, Tokyo, Japan) equipped with a HBO-100 mercury lamp and an Olympus DP73 camera. To detect the GFP signals, a GFP filter set (excitation filter 460–480, dichromatic mirror 585, emission filter 495–540) was used. For vacuolar membrane staining, the cells prepared as above were incubated with 1 μM FM4-64 (*N*-(3-triethylammoniumpropyl)-4-(6-(4-(diethylamino) phenyl) hexatrienyl) pyridinium dibromide) at 30 °C for 30 min. Cells were washed twice with MM/Gal-Ura, resuspended in fresh MM/Gal-Ura and observed under the fluorescence microscope (excitation filter 540–550, dichromatic mirror 570, emission filter 575–625). When visualizing the Ag(I)-exposed cells, the washing medium contained the corresponding concentration of AgNO_3_. The microscopic photographs were processed using the CellSens Dimension V1 imaging software (Olympus, Tokyo, Japan). For each strain, one representative image is shown.

### 2.6. Statistics

All experiments were repeated, independently, in three biological replicates at least. For each individual experiment, values were expressed as the mean ± standard error of the mean (SEM). For visual experiments, the observed trends were fully consistent among the independent experiments and a representative example is shown. The numerical data were examined by analysis of variance with multiple comparisons (ANOVA) using the statistical software Prism version 6.05 for Windows (GraphPad Software, La Jolla, CA, USA). The differences were considered to be significant when *p* < 0.05. One sample *t* test was used for the statistical analysis of each strain/condition compared with a strain/condition considered as reference. Asterisks indicate the level of significance: * *p* < 0.05, ** *p* < 0.01, and *** *p* < 0.001.

## 3. Results

### 3.1. Ag(I) Uptake by Yeast Cells Depends on Ctr1 and Is Facilitated by Cu(II) Depletion

**Figure 1:**
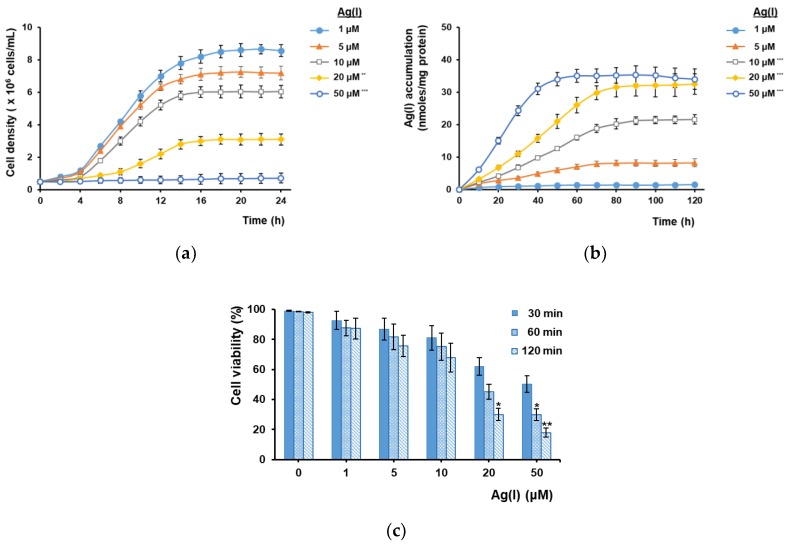
Effect of Ag(I) on *S. cerevisiae* parental cells. Exponentially growing BY4741 “wild-type” (WT) cells were inoculated in MM/Gal with various Ag(I) concentrations as described in Materials and Methods. (**a**) Effect of Ag(I) concentration on yeast growth as seen by cell proliferation. Cell growth in liquid MM/Gal was not significantly different from cell growth in the presence of 1 μM Ag(I) and for clarity reasons it is not shown. (**b**) Effect of Ag(I) concentration on Ag(I) accumulation. Total Ag(I) accumulated by cells was determined by ICP–MS and normalized to cell protein. (**c**) Cell viability of cells exposed to Ag(I). The percentage of viable cells was determined at specific time points by staining with methylene blue. One-way ANOVA (**a**,**b**) followed by Bonferroni’s test compared to low (1 μM) Ag(I). Two-way ANOVA (**c**) followed by Bonferroni’s test compared to control (no added Ag(I)). * *p* < 0.05; ** *p* < 0.01; *** *p* < 0.001.

The main target of our study was to determine whether the heterologous expression of plant MTs targeted to yeast plasma membrane results in enhanced Ag(I) accumulation, preferably without developing sensitivity to Ag(I). If heterologous MTs are anchored to the inner face of plasma membrane, it is expected that they would act as a buffer against the toxic Ag(I) ions which enter the cell. To be bound by MTs, Ag(I) must cross the plasma membrane; therefore, the capacity of parental yeast cells to accumulate Ag(I) was initially tested. Yeast growth media—even the minimal ones—have a complex composition, which includes substances that bind easily to metal cations, rendering them less toxic. This is why, in spite of the proved toxicity of Ag(I), the laboratory BY4741 strain (parental strain, “wild-type”, WT) used in our study proliferated in MM/Gal medium containing up to 10 μM AgNO_3_; even at 20 μM the cell proliferation was still noted, albeit at a lower rate (Figure 1a).

The reduction in cell proliferation could be correlated with Ag(I) accumulation: the higher the Ag(I) uptake, the more affected the cell growth (Figure 1a,b). This may explain why the rapid Ag(I) uptake from MM/Gal medium containing 50 μM AgNO_3_ resulted in almost complete arrest of cell growth (Figure 1a,b, open circles). Increasing Ag(I) concentration also affected cell viability (Figure 1c). Thus, while exposing the cells to 1–10 μM Ag(I) for up to 2 h (the time required for attaining stationary accumulation) did not significantly alter their viability, higher Ag(I) concentrations led to viability loss even after 30 min of exposure (Figure 1c). Taking into account these observations, the Ag(I) concentrations used in the subsequent experiments did not surpass 10 μM, to avoid severe toxicity.

**Figure 2:**
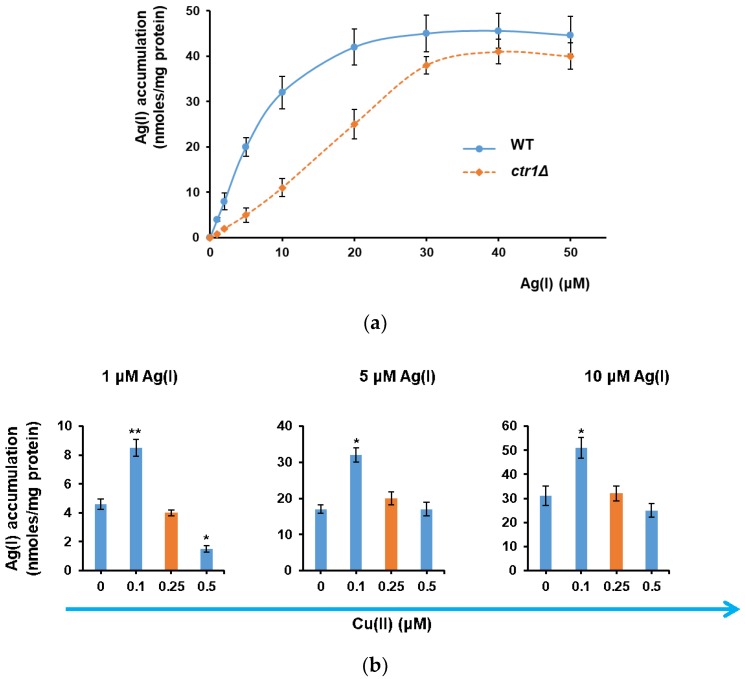
Ag(I) accumulation by *S. cerevisiae cells*. WT cells prepared as described in Materials and Methods were grown to 5 × 10^6^ cells/mL before Ag(I) was added to the specified concentration. Incubation (30 °C, 200 rpm) continued for 2 h before cells were harvested and subjected to metal assay. (**a**) Effect of *CTR1* deletion on Ag(I) accumulation. (**b**) Effect of Cu(II) depletion on Ag(I) accumulation by WT cells. Cu(II) drop-out MM was supplemented with increasing concentrations of Cu(II). The normal concentration of Cu(II) in MM is 0.25 µM (red bars). Asterisks indicate that the mean Ag(I) accumulation is significantly different from the mean Ag(I) accumulation under normal 0.25 µM Cu(II), according to one sample *t* test. * *p* < 0.05; ** *p* < 0.01.

Ag(I) is a nonessential cation resembling the essential Cu(I), with which it shares the same group of transitional metals. Copper is required for the activity of a multitude of proteins, often acting as a cofactor necessary for electron transfer reactions, due to its ability to shuttle between two oxidative states, Cu(I) and Cu(II) [24,25]. In the growth environment, copper is encountered mainly in its more stable form, Cu(II). Under low concentration conditions, corresponding to the normal growth medium, copper is carried into the yeast cell by the high affinity Cu(I) transporter Ctr1, after being reduced by cell surface reductases Fre1 and Fre2 [26,27]. To see if Ctr1 can also transport Ag(I) across the plasma membrane, Ag(I) accumulation by cells lacking Ctr1 (knockout strain *ctr1Δ*) was determined. It was noted that Ag(I) accumulation by *ctr1Δ* was significantly lower than in the case of WT cells (*p* < 0.05, one sample *t* test) up to concentrations 20–30 µM (Figure 2a). This observation indicated that that Ctr1 is involved in the transport of Ag(I) across the plasma membrane. In this line of evidence, it had been shown that the CTR1 expression is inhibited not only by high Cu(II), but also by Ag(I) [28].

At Ag(I) concentrations which were higher than 30 μM no significant difference (*p* > 0.05) between *ctr1Δ* and WT cells was noticed, indicating that other low-affinity or nonspecific transport systems may be involved. To check this possibility, it was tested if other plasma membrane transporters known to carry copper inside the cell were also involved in Ag(I) transport. In *S. cerevisiae*, Fet4 (iron transporter), Smf1 (high-affinity manganese transporter) or Pho84 (inorganic phosphate transporter) are also known to transport copper across the plasma membrane with low affinity [29,30,31]. Fet3, another transporter for Cu(II) could not be investigated as *fet3Δ* knockout cells do not grow well in synthetic media [25]. No significant difference could be noted between WT cells and strains lacking Fet3, Smf1, or Pho84 (*fet3Δ*, *smf1Δ*, or *pho84Δ* knockout strains, respectively) (Figure S1) indicating that at the concentrations used in our study, these transporter are not relevant for Ag(I) uptake.

If Ag(I) at lower concentrations is transported by Ctr1 and if Cu(I) is the natural substrate of Ctr1, then it is probable that the copper ions normally present in the growth media would compete with Ag(I) uptake via Ctr1. In yeast synthetic growth media, the normal concentration of Cu(II) is approximately 0.25 μM [18]; to be taken by Ctr1, Cu(II) is first reduced to Cu(I) by cell surface reductases Fre1 and Fre2 [26,27]. We noticed that lowering Cu(II) concentration under the value found in normal MM (i.e., 0.25 µM, red bars) significantly (*p* < 0.05) increased Ag(I) accumulation by yeast cells exposed to 1–10 μM AgNO_3_ (Figure 2b). In fact, the highest Ag(I) accumulation occurred in the presence of “traces” of Cu(II) (i.e., 0.1 μM), suggesting that a minimal presence of Cu(II) is necessary for a more robust Ag(I) uptake (Figure 2b). For this reason, to maximize the Ag(I) accumulation by yeast cells, the media used in further experiments involving Ag(I) accumulation contained 0.1 μM of Cu(II), which corresponded to approximately a quarter of the normal copper concentration used in standard media. This medium was denoted MMLC (minimal medium with low copper).

### 3.2. Ag(I) Accumulation by Yeast Cells Expressing Plant MTs Targeted to the Inner Face of Plasma Membrane

In a previous study, we reported the expression in yeast of seven *Arabidopsis thaliana* (AtMT1a, AtMT1c, AtMT2a, AtMT2b, AtMT3, AtMT4a, and AtMT4b) and four *Noccaea caerulescens* (NcMT1, NcMT2a, NcMT2b, and NcMT3) MTs, along with *S. cerevisiae* MT, Cup1 [10]. The cDNAs of the corresponding MTs had been fused to myrGFP (displaying an *N*-terminal myristoylation sequence) and expressed in *S. cerevisiae* cells under the control of an inducible promoter, *GAL1* [10]. The *GAL1* promoter allows the expression only when the cells are grown on galactose, while being repressed by the usual carbon source, glucose [19].

**Figure 3:**
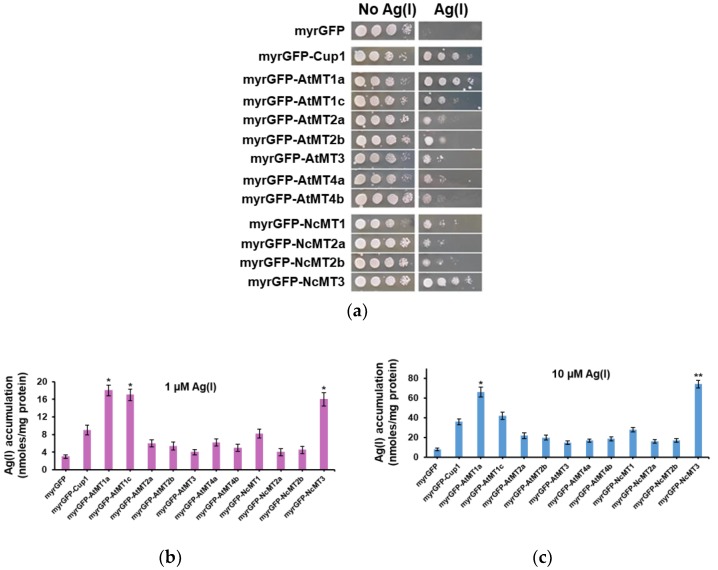
Ag(I)-related phenotype of yeast cells expressing plant MTs. The *cup2Δ* cells transformed with *URA3*-based plasmids harboring myrGFP-MT under the control of *GAL1* promoter were pregrown in SRaf-Ura then shifted to galactose-containing media for transgene induction. (**a**) Growth on solid medium containing Ag(I). Cells pregrown in SGal-Ura were 10-fold serially diluted and stamped on SGal-Ura/agar containing or not 100 μM AgNO_3_. Plates were photographed after 3 days’ incubation at 30 °C. (**b**,**c**) Ag(I) accumulation by *cup2Δ* cells expressing myrGFP-MTs. Transformed cells pregrown in MM/Raf-Ura were shifted to MMLC/Gal-Ura for transgene induction and incubated for 6 h (30 °C, 200 rpm) before Ag(I) was added at 1 μM (**b**), and 10 μM (**c**), final concentrations. Cells were further incubated with Ag(I) for 2 h before being harvested for metal assay. Asterisks indicate that the mean of the Ag(I) accumulation by a given myrGFP-MT strain is significantly different from the mean of the Ag(I) accumulation by myrGFP-Cup1 under the same conditions, according to one sample *t* test. * *p* < 0.05; ** *p* < 0.01.

The *S. cerevisiae* native MT, Cup1, is a Cu(I)-thionein [11,12,24] whose expression is strongly induced by Cu(II) [32] but also by Ag(I) [28]. To avoid interference with the innate MT, using cells defective in Cup1 was indicated. Nevertheless, knocking out the *CUP1* gene is complicated by the fact that the *CUP1* locus has multiple copies, being variably amplified in different yeast strains [33]. As *CUP1* transcription is specifically induced by the copper-dependent transcription activator Cup2 [32,34,35], the heterologous expression of plant MTs was done in the yeast knockout mutant *cup2Δ*, thus eluding the native *CUP1* transcription. Preliminary results indicated that the expression of all plant MTs tested augmented the tolerance to Ag(I) of *cup2Δ* cells (Figure 3a), indicating that the recombinant MTs were active in alleviating the Ag(I) toxicity towards yeast cells. Considering the growth on solid medium containing Ag(I), *cup2Δ* cells expressing myrGFP-Cup1 and myrGFP-NcMT3 showed the most robust growth in the presence of surplus Ag(I), followed by myrGFP-AtMT1a and myrGFP-AtMT1c (Figure 3a). These results were paralleled by Ag(I) LC_50_ (the concentration which reduced cell proliferation by half after 24 h of exposure to the metal): thus, while for *cup2Δ* cells expressing myrGFP the average LC_50_ was 17.6 µM, for *cup2Δ* cells expressing myrGFP-AtMT1a and myrGFP-NcMT3 the average values for LC_50_ were considerably higher (39.5 μM and 54.6 μM, respectively). In all strains tested which expressed transgenic MTs, the LC_50_ was higher than for cells expressing the control myrGFP (Table S1); this observation indicated that heterologous expression of MT cDNA results in increased tolerance of yeast cells to Ag(I).

Ag(I) accumulation by *cup2Δ* cells expressing plant MTs was further determined. Under low concentration conditions (1 μM), *cup2Δ* cells expressing myrGFP-Cup1, myrGFP-AtMT1a, myrGFP-AtMT1c, and myrGFP-NcMT3 clearly accumulated Ag(I); moreover, the cells expressing the three plant MTs mentioned above accumulated significantly more than the cells expressing the yeast Cup1 (Figure 3b). Under high Ag(I) (10 μM), all transformants accumulated more Ag(I) compared to control myrGFP. Cells expressing myrGFP-AtMT1a and myrGFP-NcMT3 accumulated significantly more Ag(I) than myrGFP-Cup1 (Figure 3c). The strains exhibiting enhanced Ag(I) accumulation were also rendered more fit by the corresponding MT expression (data not shown) suggesting that the higher Ag(I) accumulation may be the result of robust strain growth rather than MT specificity for Ag(I).

**Figure 4:**
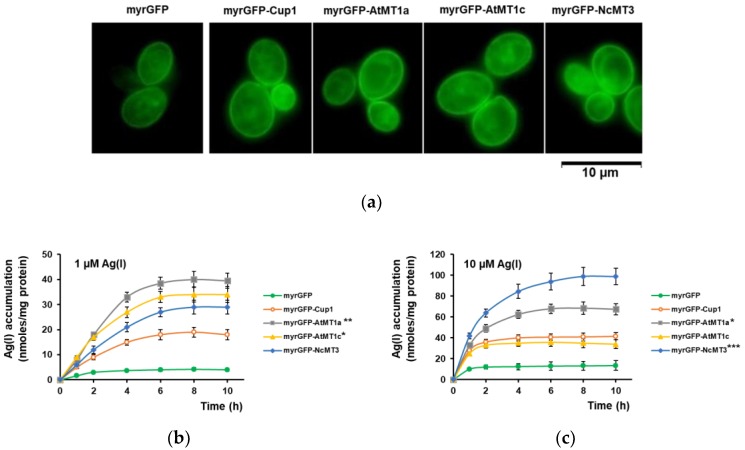
Ag(I) accumulation by selected strains. The *cup2Δ* cells transformed with *URA3*-based plasmids harboring myrGFP (control), myrGFP-Cup1 (yeast MT), myrGFP-AtMT1a, myrGFP-AtMT1c and myrGFP-NcMT3 under the control of *GAL1* promoter were pregrown in MM/Raf-Ura then shifted to galactose-containing medium for transgene induction. (**a**) Cellular localization of myrGFP-MTx. Transgenic cells were shifted to MM/Gal-Ura. Live cells were visualized by fluorescence microscopy using a GFP filter set. Images shown were taken 6 h after the galactose shift. (**b**,**c**) Accumulation of Ag(I) over time. Transformed cells pregrown in MM/Raf-Ura were shifted to MMLC/Gal-Ura and incubated 6 h for transgene induction (30 °C, 200 rpm) before Ag(I) was added at 1 μM (**b**), and 10 μM (**c**), final concentrations. Cells were incubated with Ag(I) for various times and harvested for metal assay. One-way ANOVA (**b**,**c**) followed by Bonferroni’s test compared to myrGFP-Cup1. * *p* < 0.05; ** *p* < 0.01; *** *p* < 0.001.

The yeast strains expressing myrGFP-Cup1, myrGFP-AtMT1a, myrGFP-AtMT1c, and myrGFP-NcMT3 were investigated further under both low Ag(I) (1 μM) and high Ag(I) (10 μM, as the highest concentration which did not significantly affect the viability of the mutant cells, data not shown). All the strains tested exhibited green fluorescence at the plasma membrane level, indicating that the myrGFP-MT were not only expressed but also targeted to the plasma membrane of the *cup2Δ* cells (Figure 4a). Under 1 μM Ag(I), all mutant strains accumulated more Ag(I) than the myrGFP control strain, attaining a saturation plateau after 4–6 h of exposure. Remarkably, the strains expressing plant MTs accumulated more Ag(I) than the strain expressing myrGFP-Cup1, with myrGFP-AtMT1a being the most efficient accumulator (Figure 4b). Under high concentration (10 μM), the Ag(I) accumulation by the strains expressing MTs became more rapid, with a plateau reached in the first 2–4 h of exposure. Yeast cells expressing myrGFP-NcMT3 were the best accumulators: Ag(I) accumulation continued even after 4 h, to reach a plateau after 8–10 h of Ag(I) exposure (Figure 4c). These cells were also the most tolerant to Ag(I): thus, more than 75% and 50% of cells expressing myrGFP-NcMT3 were still viable after 8 h exposure to 25 μM and 50 μM, respectively (data not shown).

**Figure 5:**
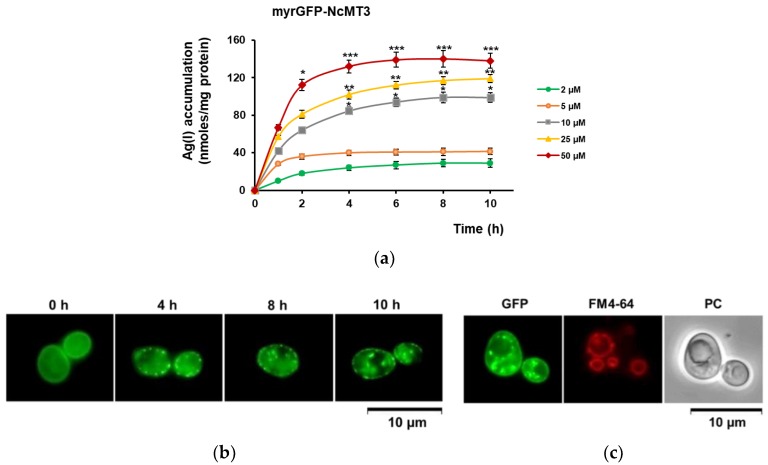
Ag(I) accumulation by the Ag(I)-tolerant strain myrGFP-NcMT3. The *cup2Δ* cells transformed with *URA3*-based plasmid harboring myrGFP-NcMT3 under the control of *GAL1* promoter were pregrown in MM/Raf-Ura, then shifted to galactose medium for transgene induction. (**a**) Effect of Ag(I) concentration on Ag(I) accumulation. Transformed cells pregrown in MM/Raf-Ura were shifted to MMLC/Gal-Ura and incubated 6 h for transgene induction (30 °C, 200 rpm) before Ag(I) was added to the specified concentrations. Cells were incubated with Ag(I) for various times and harvested for metal assay. Two-way ANOVA followed by Bonferroni’s test compared to the data obtained for 1 µM Ag(I) (shown in Figure 3b). * *p* < 0.05; ** *p* < 0.01; *** *p* < 0.001. (**b**) Effect of high Ag(I) on myrGFP-NcMT localization. Cells incubated with 25 μM Ag(I) were harvested at various times for myrGFP-NcMT3 visualization by fluorescence microscopy. (**c**) Localization of myrGFP-NcMT3 at the vacuolar membrane. Cells expressing myrGFP-NcMT3 exposed for 10 h to 25 μM Ag(I) were incubated with FM4-64 for vacuole membrane straining. Cells were visualized by fluorescence microscopy (λ_excit_ 460–480 nm, λ_emiss_ 495–540 nm for GFP; λ_excit_ 540–550 nm, λ_emiss_ 575–625 nm for FM4-64). PC = Phase Contrast.

Yeast cells expressing myrGFP-NcMT3 accumulated Ag(I) from the medium in a concentration-dependent manner (Figure 5a).

It was noticed that when exposed to higher Ag(I) (25–50 μM), myrGFP-NcMT3 no longer exhibited the continuous localization pattern at the plasma membrane; instead, conglomerate fluorescent patches were observed (Figure 5b). These conglomerates could be seen both at the cell periphery and also surrounding the vacuoles as revealed by staining with the vacuolar tracer FM4-64 (Figure 5c). This observation suggested the possibility that Ag(I) ions bind to the anchored myrGFP-NcMT3 inducing MT agglutination [36] and endosome-to-vacuole-like traffic [37] for metal-MT homeostasis, as is the case of “cadmosomes” [38]. Binding of Ag(I) to MTs lowers the concentration of free Ag(I) ions, and subsequently their toxicity.

### 3.3. Accumulation of Ultra-Trace Ag(I)

**Figure 6:**
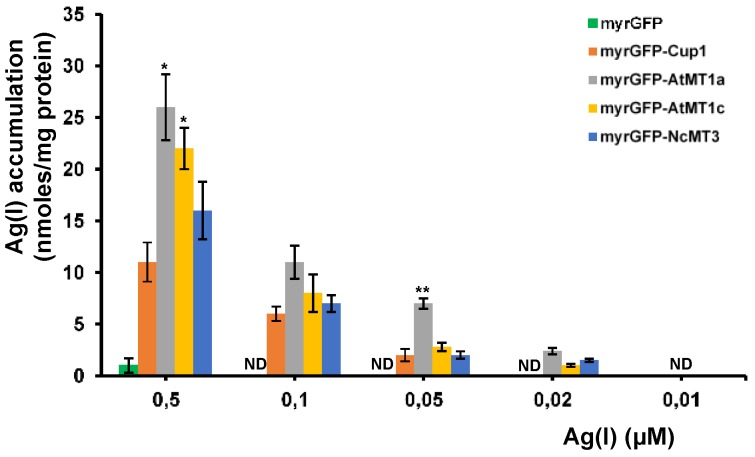
Ag(I) accumulation from media containing trace Ag(I) concentrations. The *cup2Δ* cells transformed with *URA3*-based plasmids harboring myrGFP (control), myrGFP-Cup1 (ScMT), myrGFP-AtMT1a, myrGFP-AtMT1c, and myrGFP-NcMT3 under the control of *GAL1* promoter were pregrown in MMLC/Raf-Ura then shifted to MMLC/Gal-Ura for transgene induction. After 6 h, cells were shifted to MMLC/Gal-Ura containing low concentrations of Ag(I). Metal accumulation was assayed after 10 h incubation (30 °C, 200 rpm. Two-way ANOVA followed by Bonferroni’s test compared to control myrGFP. * *p* < 0.05; ** *p* < 0.01.

Heavy metal accumulating organisms can be of value when having the capacity to remove the surplus cations from contaminated sites without developing toxicity symptoms, at the same time producing sufficient biomass to be of biotechnical relevance. Another desired trait for biotechnology is the capacity of an organism to take up and accumulate a compound from environments containing (ultra)trace amounts of the substance of interest. We therefore tested the capacity of the yeast cells expressing myrGFP-Cup1, myrGFP-AtMT1a, myrGFP-AtMT1c, and myrGFP-NcMT3 to accumulate Ag(I) from MMLC supplemented with “trace” concentrations of AgNO_3_ (0.5–0.01 μM). It was noted that all strains accumulated more Ag(I) than control myrGFP; the strain expressing myrGFP-AtMT1a accumulated significantly more than strain myrGFP-Cup1 expressing yeast MT (Figure 6). The Ag(I) accumulation by recombinant strains could be recorded at Ag(I) as low as 0.02 μM (Figure 6).

## 4. Discussion

Among the natural heavy metal accumulators known [39,40,41,42], there are few examples of Ag(I) accumulating organisms described [43,44,45,46], and even less with biotechnology potential [47]. In the present study it was aimed to obtain Ag(I) accumulating cells by heterologous expression of plant MTs targeted to the inner face of yeast plasma membrane. Out of the eleven transgenic yeast strains expressing plant MTs, two showed remarkable properties. The strain expressing myrGFP-NcMT3 showed both Ag(I) tolerance and increased capacity to accumulate Ag(I) from high-concentration media. The strain expressing myrGFP-AtMT1a also had the capacity to accumulate Ag(I) from media which contained only traces of this metal. This observation is relevant, as such strains could be used for concentrating heavy metals from effluents which contain only traces of such elements, whose presence represent a hazard even when their concentration is within the accepted limits. Taken together, these findings support the importance of the two strains for Ag(I)-related biotechnologies such as bioremediation, bioaccumulation, and bioconcentration. Importantly, the experiments reported here have been done with MT genes cloned in plasmids, with auxotrophic mutants and in synthetic medium growth conditions. As these are not the best conditions for use of industrial strains, the extrapolation of our experiments by using integrative constructions and using promoters which induce strong expression under less stringent conditions represent a desiderate for extending our findings to biotechnology applications.

## Supplementary Materials

The following are available online at http://www.mdpi.com/2073-4409/7/12/266/s1, Figure S1: Ag(I) accumulation by *S. cerevisiae* cells lacking low affinity copper transporters. Table S1: Ag(I) toxicity (LC_50_) towards *S. cerevisiae* strains overexpressing plant MTs.

## References

[1] Johnson D.B. (2013). Development and application of biotechnologies in the metal mining industry. Environ. Sci. Pollut. Res. Int..

[2] Ratte H.T. (1999). Bioaccumulation and toxicity of silver compounds: A review. Environ. Toxicol. Chem..

[3] Zhang W., Xiao B., Fang T. (2018). Chemical transformation of silver nanoparticles in aquatic environments: Mechanism, morphology and toxicity. Chemosphere.

[4] Purcell T.W., Peters J.J. (1998). Sources of silver in the environment. Environ. Toxicol. Chem..

[5] Kuroda K., Ueda M. (2010). Engineering of microorganisms towards recovery of rare metal ions. Appl. Microbiol. Biotechnol..

[6] Soares E.V., Soares H.M. (2013). Cleanup of industrial effluents containing heavy metals: A new opportunity of valorising the biomass produced by brewing industry. Appl. Microbiol. Biotechnol..

[7] Liu Z., Ho S.H., Hasunuma T., Chang J.S., Ren N.Q., Kondo A. (2016). Recent advances in yeast cell-surface display technologies for waste biorefineries. Bioresour. Technol..

[8] Liang X., Gadd G.M. (2017). Metal and metalloid biorecovery using fungi. Microb. Biotechnol..

[9] Ruta L.L., Kissen R., Nicolau I., Neagoe A.D., Petrescu A.J., Bones A.M., Farcasanu I.C. (2017). Heavy metal accumulation by *Saccharomyces cerevisiae* cells armed with metal binding hexapeptides targeted to the inner face of the plasma membrane. Appl. Microbiol. Biotechnol..

[10] Ruta L.L., Lin Y.F., Kissen R., Nicolau I., Neagoe A.D., Ghenea S., Bones A.M., Farcasanu I.C. (2017). Anchoring plant metallothioneins to the inner face of the plasma membrane of *Saccharomyces cerevisiae* cells leads to heavy metal accumulation. PLoS ONE.

[11] Capdevila M., Atrian S. (2011). Metallothionein protein evolution: A miniassay. J. Biol. Inorg. Chem..

[12] Palacios O., Atrian S., Capdevila M. (2011). Zn- and Cu-thioneins: A functional classification for metallothioneins?. J. Biol. Inorg. Chem..

[13] Valls M., Bofill R., Gonzalez-Duarte R., Gonzalez-Duarte P., Capdevila M., Atrian S. (2001). A new insight into metallothionein (MT) classification and evolution: The in vivo and in vitro metal binding features of *Homarus americanus* recombinant MT. J. Biol. Chem..

[14] Vasak M., Riordan J.F., Vallee B.L. (1991). Metal removal and substitution in vertebrate and invertebrate metallothioneins. Metallobiochemistry Part. B, Metallothionein and Related Molecules.

[15] Farcasanu I.C., Ruta L.L., Lucas C., Pais C. (2017). Metallothioneins, *Saccharomyces cerevisiae*, and heavy metals: A biotechnology triad?. Old Yeasts, New Questions.

[16] Zhang M., Takano T., Liu S., Zhang X. (2014). Abiotic stress response in yeast and metal-binding ability of a type 2 metallothionein-like protein (PutMT2) from *Puccinellia tenuiflora*. Mol. Biol Rep..

[17] Brachmann C.B., Davies A., Cost G.J., Caputo E., Li J., Hieter P., Boeke J.D. (1998). Designer deletion strains derived from *Saccharomyces* cerevisiae S288C: A useful set of strains and plasmids for PCR-mediated gene disruption and other applications. Yeast.

[18] Sherman F. (2002). Getting started with yeast. Methods Enzymol..

[19] Guthrie C., Fink G.R. (1991). Guide to yeast genetics and molecular biology. Methods Enzymol..

[20] Cohen R., Engelberg D. (2007). Commonly used *Saccharomyces cerevisiae* strains (e.g. BY4741, W303) are growth sensitive on synthetic complete medium due to poor leucine uptake. FEMS Microbiol. Lett..

[21] Dohmen R.J., Strasser A.W.M., Honer C.B., Hollenberg C.P. (1991). An efficient transformation procedure enabling long-term storage of competent cells of various yeast genera. Yeast.

[22] Amberg D.C., Burke D.J., Strathern J.N., Burke D., Dawson D., Stearns T. (2005). Measuring yeast cell density by spectrophotometry. Methods in Yeast Genetics. A Cold Spring Harbor Laboratory Course Manual.

[23] Bradford M.M. (1976). A rapid and sensitive method for the quantitation of microgram quantities of protein utilizing the principle of protein-dye binding. Anal. Biochem..

[24] Jensen L.T., Howard W.R., Strain J.J., Winge D.R., Culotta V.C. (1996). Enhanced effectiveness of copper ion buffering by CUP1 metallothionein compared with CRS5 metallothionein in *Saccharomyces cerevisiae*. J. Biol. Chem..

[25] De Freitas J., Wintz H., Kim J.H., Poynton H., Fox T., Vulpe C. (2003). A model organism for iron and copper metabolism studies. Biometals.

[26] Shi X., Stoj C., Romeo A., Kosman D.J., Zhu Z. (2003). Fre1p Cu^2+^ reduction and Fet3p Cu^1+^ oxidation modulate copper toxicity in *Saccharomyces cerevisiae*. J. Biol. Chem..

[27] Nevitt T., Ohrvik H., Thiele D.J. (2012). Charting the travels of copper in eukaryotes from yeast to mammals. Biochim. Biophys. Acta.

[28] Niazi J.H., Sang B.I., Kim Y.S., Gu M.B. (2011). Global gene response in *Saccharomyces cerevisiae* exposed to silver nanoparticles. Appl. Biochem. Biotechnol..

[29] Hassett R., Dix D.R., Eide D.J., Kosman D.J. (2000). The Fe(II) permease Fet4p functions as a low affinity copper transporter and supports normal copper trafficking in *Saccharomyces cerevisiae*. Biochem. J..

[30] Cohen A., Nelson H., Nelson N. (2000). The family of SMF metal ion transporters in yeast cells. J. Biol. Chem..

[31] Jensen L.T., Ajua-Alemanji M., Culotta V.C. (2003). The *Saccharomyces cerevisiae* high affinity phosphate transporter encoded by *PHO84* also functions in manganese homeostasis. J. Biol. Chem..

[32] Thiele D.J. (1988). *ACE1* regulates expression of the *Saccharomyces cerevisiae* metallothionein gene. Mol. Cell. Biol..

[33] Zhao Y., Strope P.K., Kozmin S.G., McCusker J.H., Dietrich F.S., Kokoska R.J., Petes T.D. (2014). Structures of naturally evolved *CUP1* tandem arrays in yeast indicate that these arrays are generated by unequal nonhomologous recombination. G3 (Bethesda).

[34] Welch J., Fogel S., Buchman C., Karin M. (1989). The *CUP2* gene product regulates the expression of the *CUP1* gene, coding for yeast metallothionein. EMBO J..

[35] Buchman C., Skroch P., Welch J., Fogel S., Karin M. (1989). The *CUP2* gene product, regulator of yeast metallothionein expression, is a copper-activated DNA-binding protein. Mol. Cell. Biol..

[36] Capdevila M., Bofilla R., Palaciosa O., Atrian S. (2011). State-of-the-art of metallothioneins at the beginning of the 21st century. Coord Chem. Rev..

[37] Hayden J., Williams M., Granich A., Ahn H., Tenay B., Lukehart J., Highfill C., Dobard S., Kim K. (2013). Vps1 in the late endosome-to-vacuole traffic. J. Biosci..

[38] Orihuela R., Domènech J., Bofill R., You C., Mackay E.A., Kägi J.H., Capdevila M., Atrian S. (2008). The metal-binding features of the recombinant mussel *Mytilus edulis* MT-10-IV metallothionein. J. Biol. Inorg. Chem..

[39] Krzciuk K., Gałuszka A. (2015). Prospecting for hyperaccumulators of trace elements: A review. Crit. Rev. Biotechnol..

[40] Pollard A.J., Reeves R.D., Baker A.J. (2014). Facultative hyperaccumulation of heavy metals and metalloids. Plant Sci..

[41] Miransari M. (2011). Hyperaccumulators, arbuscular mycorrhizal fungi and stress of heavy metals. Biotechnol. Adv..

[42] Gifford S., Dunstan R.H., O’Connor W., Koller C.E., MacFarlane G.R. (2007). Aquatic zooremediation: Deploying animals to remediate contaminated aquatic environments. Trends Biotechnol..

[43] Borovicka J., Kotrba P., Gryndler M., Mihaljevic M., Randa Z., Rohovec J., Cajthaml T., Stijve T., Dunn C.E. (2010). Bioaccumulation of silver in ectomycorrhizal and saprobic macrofungi from pristine and polluted areas. Sci. Total Environ..

[44] Yang W., Li H., Zhang T., Sen L., Ni W. (2014). Classification and identification of metal-accumulating plant species by cluster analysis. Environ. Sci. Pollut. Res. Int..

[45] Saavedra Y., González A., Fernández P., Blanco J. (2004). Interspecific variation of metal concentrations in three bivalve mollusks from Galicia. Arch. Environ. Contam. Toxicol..

[46] Borovicka J., Randa Z., Jelínek E., Kotrba P., Dunn C.E. (2007). Hyperaccumulation of silver by *amanita strobiliformis* and related species of the section *Lepidella*. Mycol. Res..

[47] Haverkamp R.G., Marshall A.T., van Agterveld D. (2007). Pick your carats: Nanoparticles of gold-silver-copper alloy produced in vivo. J. Nanopart. Res..

