# Supplementary material for: Accumulation of Ag(I) by Saccharomyces cerevisiae Cells Expressing Plant Metallothioneins
Source: Cells. 2018 Dec 11;7(12):266. doi: 10.3390/cells7120266 (PMC6315939; doi:10.3390/cells7120266)
Supplement: Supplementary file 1 [file cells-07-00266-s001.pdf]

cells

MDPI

Supplementary Files

Accumulation of Ag(I) by *Saccharomyces cerevisiae* Cells Expressing Plant Metallothioneins

Lavinia L. Ruta [1], Melania A. Banu [2¶], Aurora D. Neagoe [2], Ralph Kissen [3], Atle M. Bones [3], and Ileana C. Farcasanu [1,*]

[1] Faculty of Chemistry, University of Bucharest, Sos. Panduri 90-92, 050663 Bucharest, Romania; lavinia.ruta@chimie.unibuc.ro (L.L.R.)
[2] Faculty of Biology, University of Bucharest, Splaiul Independentei 91-95, 050095 Bucharest, Romania; melania.banu@imt.ro (M.A.B.); auroradaniela.neagoe@g.unibuc.ro (A.D.N.)
[3] Cell, Molecular Biology and Genomics Group, Department of Biology, Norwegian University of Science and Technology, NO-7491 Trondheim, Norway; ralph.kissen@ntnu.no (R.K.); atle.bones@bio.ntnu.no (A.M.B.)
¶ Present address: National Institute for Research and Development in Microtechnologies – IMT Bucharest, Erou Iancu Nicolae 126A, 077190 Bucharest, Romania
* Correspondence: ileana.farcasanu@chimie.unibuc.ro; Tel.: +40-721-067-169

Received: 13 November 2018; Accepted: 10 December 2018; Published: 11 December 2018

Abstract: The various applications of Ag(I) generated the necessity to obtain Ag(I)-accumulating organisms for the removal of surplus Ag(I) from contaminated sites or for the concentration of Ag(I) from Ag(I)-poor environments. In this study we obtained Ag(I)-accumulating cells by expressing plant metallothioneins (MTs) in the model *Saccharomyces cerevisiae*. The cDNAs of seven *Arabidopsis thaliana* MTs (AtMT1a, AtMT1c, AtMT2a, AtMT2b, AtMT3, AtMT4a and AtMT4b) and four *Noccaea caerulescens* MTs (NcMT1, NcMT2a, NcMT2b and NcMT3) fused to myrGFP displaying an N-terminal myristoylation sequence for plasma membrane targeting were expressed in *S. cerevisiae* and checked for Ag(I)-related phenotype. The transgenic yeast cells were grown in copper-deficient media to ensure the expression of the plasma membrane high-affinity Cu(I) transporter Ctr1, and also to elude the copper-related inhibition of Ag(I) transport into the cell. All plant MTs expressed in *S. cerevisiae* conferred Ag(I) tolerance to the yeast cells. Among them, myrGFP-NcMT3 afforded Ag(I) accumulation under high concentration (10-50 μM), while myrGFP-AtMT1a conferred increased accumulation capacity under low (1 μM) or even trace Ag(I) (0.02-0.05 μM). The ability to tolerate high concentrations of Ag(I) coupled with accumulative characteristics and robust growth showed by some of the transgenic yeasts highlighted the potential of these strains for biotechnology applications.

Keywords: silver; metallothionein; *Arabidopsis thaliana*; *Noccaea caerulescens*; *Saccharomyces cerevisiae*; accumulation

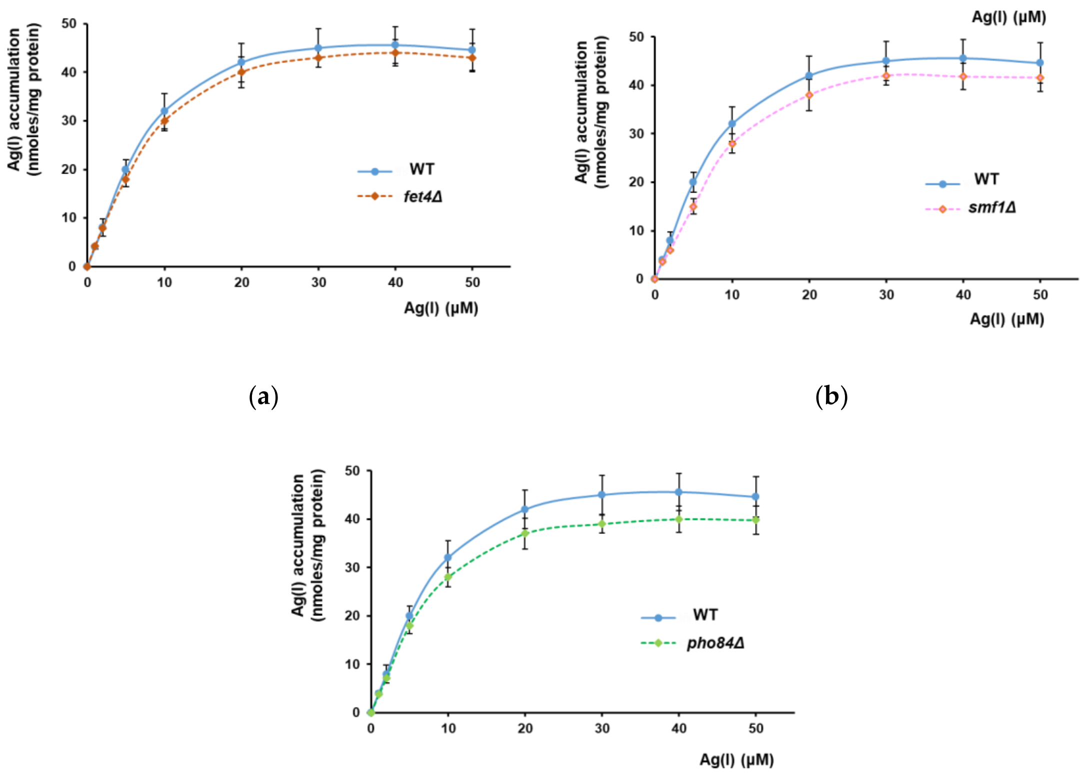

(**a**) (**b**)

(**c**)

Figure S1. Ag(I) accumulation by *S. cerevisiae* cells with defects in low affinity copper transporters. Cells prepared as described in Materials and Methods were grown to 5 × 10^6 cells/mL before Ag(I) was added to the specified concentration. Incubation (30 °C, 200 rpm) continued for 2 h before cells were harvested and subjected to metal assay. (**a**) Effect of *FET4* deletion on Ag(I) accumulation. (**b**) Effect of *SMF1* deletion on Ag(I) accumulation. (**c**) Effect of *PHO84* deletion on Ag(I) accumulation. Ag(I) accumulation was not significantly different in any of the four strains tested ($p > 0.05$, One-way ANOVA followed by Bonferroni's test compared to WT).

Table S1. Ag(I) toxicity (LC_{50}) towards *S. cerevisiae* strains overexpressing plant MTs. LC_{50} was determined as the the Ag(I) concentration eliciting a 50% reduction in cell proliferation after 24 hours of incubation (30 °C, 200 rpm) with various Ag(I) concentrations.

Strain	LC_{50} (μM)
WT [1]	16.5 ± 2.2
myrGFP	17.6 ± 1.9
myrGFP-Cup1	28.4 ±3.2
myrGFP-AtMT1a	39.5 ± 7,8
myrGFP-AtMT1c	28.2 ±6.2
myrGFP-AtMT2a	24.5 ± 7.1
myrGFP-AtMT2b	23.4 ± 6.5
myrGFP-AtMT3	27.4 ± 4.6
myrGFP-AtMT4a	23.4 ± 3.8
myrGFP-AtMT4b	25.1 ± 7.8
myrGFP-NcMT1	28.3 ± 6.8
myrGFP-NcMT2a	24.2 ± 4.8
myrGFP-NcMT2b	25.3 ± 6.3
myrGFP-NcMT3	54.6 ± 8.6

[1] WT = parental strain BY4741; all the other strains were *cup2Δ* transformed with the plasmids containing the corresponding MT cDNAs.

© 2018 by the authors. Licensee MDPI, Basel, Switzerland. This article is an open access article distributed under the terms and conditions of the Creative Commons Attribution (CC BY) license (http://creativecommons.org/licenses/by/4.0/).
